# Estimating the relative fitness of escaped farmed salmon offspring in the wild and modelling the consequences of invasion for wild populations

**DOI:** 10.1111/eva.12746

**Published:** 2019-01-28

**Authors:** Emma V. A. Sylvester, Brendan F. Wringe, Steven J. Duffy, Lorraine C. Hamilton, Ian A. Fleming, Marco Castellani, Paul Bentzen, Ian R. Bradbury

**Affiliations:** ^1^ Science Branch, Fisheries and Oceans Canada St. John’s Newfoundland and Labrador Canada; ^2^ Science Branch, Department of Fisheries and Oceans Canada Bedford Institute of Oceanography Dartmouth Nova Scotia Canada; ^3^ Aquatic Biotechnology Laboratory, Fisheries and Oceans Canada Bedford Institute of Oceanography Dartmouth Nova Scotia Canada; ^4^ Memorial University of Newfoundland Department of Ocean Sciences St. John’s Newfoundland and Labrador Canada; ^5^ Department of Mechanical Engineering University of Birmingham Birmingham UK; ^6^ Marine Gene Probe Laboratory, Department of Biology Dalhousie University Halifax Nova Scotia Canada

**Keywords:** aquaculture impacts, fish farming, introgression, population eco‐genetic modelling, relative fitness, *Salmo salar*

## Abstract

Throughout their native range, wild Atlantic salmon populations are threatened by hybridization and introgression with escapees from net‐pen salmon aquaculture. Although domestic–wild hybrid offspring have shown reduced fitness in laboratory and field experiments, consequential impacts on population abundance and genetic integrity remain difficult to predict in the field, in part because the strength of selection against domestic offspring is often unknown and context‐dependent. Here, we follow a single large escape event of farmed Atlantic salmon in southern Newfoundland and monitor changes in the in‐river proportions of hybrids and feral individuals over time using genetically based hybrid identification. Over a three‐year period following the escape, the overall proportion of wild parr increased consistently (total wild proportion of 71.6%, 75.1% and 87.5% each year, respectively), with subsequent declines in feral (genetically pure farmed individuals originating from escaped, farmed adults) and hybrid parr. We quantify the strength of selection against parr of aquaculture ancestry and explore the genetic and demographic consequences for populations in the region. Within‐cohort changes in the relative proportions of feral and F1 parr suggest reduced relative survival compared to wild individuals over the first (0.15 and 0.81 for feral and F1, respectively) and second years of life (0.26, 0.83). These relative survivorship estimates were used to inform an individual‐based salmon eco‐genetic model to project changes in adult abundance and overall allele frequency across three invasion scenarios ranging from short‐term to long‐term invasion and three relative survival scenarios. Modelling results indicate that total population abundance and time to recovery were greatly affected by relative survivorship and predict significant declines in wild population abundance under continued large escape events and calculated survivorship. Overall, this work demonstrates the importance of estimating the strength of selection against domestic offspring in the wild to predict the long‐term impact of farmed salmon escape events on wild populations.

## 1 INTRODUCTION

The threat of invasion from domesticated Atlantic salmon (*Salmo salar*) into wild populations is of growing concern to management and conservation efforts (Clifford, McGinnity, & Ferguson, 1998a, 1998b; Forseth et al., 2017; Glover et al., 2012; Gross, 1998; Le Cam, Perrier, Besnard, Bernatchez, & Evanno, 2015). Farmed escapees often outnumber wild populations annually, and hybridization and genetic introgression between farmed and wild salmon have been detected throughout their native range (Bourret, O'Reilly, Carr, Berg, & Bernatchez, 2011; Glover et al., 2017). Mating of farmed and wild salmon may result in reduced genetic integrity of the wild population (Fleming et al., 2000; McGinnity et al.., 2003; Skaala et al., 2012; Solberg, Dyrhovden, Matre, & Glover, 2016) and, under pressure from continual invasion, a loss of overall population fitness (Baskett, Burgess, & Waples, 2013; McGinnity et al., 2003). The degree of genetic impact on wild populations due to invasion is often population‐specific (Baskett et al., 2013; Karlsson, Diserud, Fiske, & Hindar, 2016) and may be highly dependent on the selective pressures acting on invading individuals and their progeny (Thurman & Barrett, 2016).

Current methods of estimating these selective pressures or relative fitness of aquaculture offspring (i.e., hybrid and feral) under wild conditions are often family‐specific (Skaala et al., 2012) and rely on laborious experimental approaches (McGinnity et al., 2003, 1997; Miller, Close, & Kapuscinski, 2004). Field experiments suggest that the relative fitness of hybrid and feral individuals may follow a pattern of additive genetic inheritance (Einum & Fleming, 1997; Fleming et al., 2000; McGinnity et al., 2003), although maternal environmental effects are potentially also influential in early life stages (Houde, Black, Wilson, Pitcher, & Neff, 2015). Due to the complexity of interactions and effects on individual fitness, estimating the strength of selection at the population or regional scale remains difficult. Namely, hybridization success and selection pressures can widely vary across even small spatial scales (Sylvester et al., 2018), and controlled experiments (Skaala et al., 2012) may not reflect the conditions of wild populations and landscapes (Fleming et al., 2000). Also, the impacts of invasion by farmed individuals have been shown to vary depending on the demography of the native population (Heino, Svåsand, Wennevik, & Glover, 2015; Wringe, Jeffery, et al., 2018) and the degree of relatedness between farmed salmon and the wild populations they invade (Baskett et al., 2013). Similarly, wild individuals straying from nearby rivers may buffer the impact of domesticated invasion in populations (Castellani et al., 2018). Given this inherent complexity, enhanced understanding of the relative fitness of domestic offspring at the population level in a range of natural environments is required to better predict and mitigate impacts of escaped farmed salmon on wild populations.

Here, we capitalize on a large escape event that occurred in 2013 in southern Newfoundland to explore how these changes may be monitored and applied to understand long‐term consequences for wild populations. This single event resulted in the escape of 20,000 adult farmed salmon into a region supporting an approximately equal number of wild salmon. Previous work has documented widespread hybridization between wild and farmed escaped salmon following this escape event (Wringe, Jeffery, et al., 2018). By observing temporal changes in hybrid class composition after an influx of invaders into a system, the strength of selection against aquaculture‐derived individuals may be directly estimated for a real‐world system of invasion. As such, we aim to (a) monitor the changes in the proportion of wild, hybrid and feral parr over time, (b) use these data to estimate survivorship as a proxy of the strength of selection against feral and hybrid offspring, and (c) using these realistic estimates of selection, model the consequences for these populations over various invasion scenarios, exploring the sensitivity to the strength of selection. We build directly on previous work which developed genetic and analytical tools to identify hybrids (Anderson & Thompson, 2002; Wringe, Stanley, Jeffery, Anderson, & Bradbury, 2017a, 2017b; Wringe, Stanley, et al., 2018) and documented interbreeding between escaped farmed and wild salmon following this escape event (Wringe, Jeffery, et al., 2018). We expand on these studies and others (Clifford, McGinnity, & Ferguson, 1998a, 1998b; Fleming et al., 2000; McGinnity et al., 2003; Skaala et al., 2012) by estimating the strength of selection against domestic and hybrid offspring in the wild, and explore the importance of obtaining accurate estimates of relative survival for predicting long‐term consequences of invasion.

## 2 MATERIALS AND METHODS

### 2.1 Sampling and genotyping

**Figure 1:**
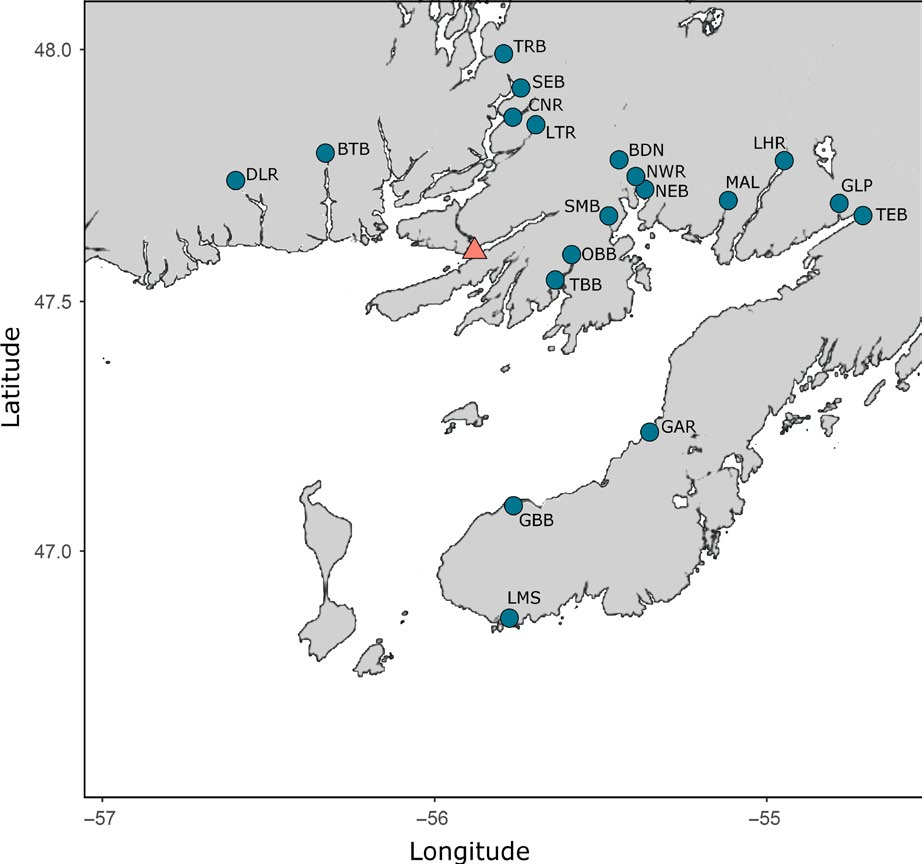
Sites in southern Newfoundland sampled in 2014–2016. Sample sizes per site and year can be found in Table 1. The location of the 2013 escape event is indicated by the pink triangle

A total of 4,619 parr were collected by electrofishing across 19 rivers in southern Newfoundland, Canada (Figure 1), in the summers of 2014, 2015 and 2016. As emergence of alevins in southern Newfoundland generally occurs in early June, summer sampling allows for collection of newly emerged individuals, that is, individuals from the previous spawning season or young‐of‐year (YoY), as well as parr remaining in streams from earlier spawning seasons, generally up to 2–4 years in Newfoundland (Porter, 1975). Individuals were assigned to an age class based on length (YoY: 0–70 mm, 1+: 71–110 mm, 2+: >110 mm) and stored in 95% ethanol for later DNA extraction and analysis. In addition to these samples, 301 wild individuals (previously identified as pure wild with high certainty) and 156 farmed reference individuals were analysed as baseline samples. Farmed references were provided from three cage sites within Newfoundland and are likely representative of escapees sampled throughout the region as salmon cages in Atlantic Canada are presently stocked only with individuals from a single, non‐local Saint John River population.

**Table 1:** Sample size per sampling year and age class for each river, after filtering at a minimum posterior probability threshold in NewHybrids. Relative survivorship was estimated from a single cohort spanning all three years (2014 young‐of‐year (YoY), 2015 1+ and 2016 2+; see Methods)

River	2014	2015		2016		
	YoY	YoY	1+	YoY	1+	2+
BDN	0	49	60	45	51	16
BTB	16	3	17	29	1	22
CNR	364	19	0	77	26	3
DLR	18	20	20	54	15	10
GAR	193	52	50	96	109	21
GBB	39	15	25	3	75	26
GLP	102	9	51	4	34	2
LHR	124	41	84	44	72	3
LTR	120	0	0	68	23	6
LMS	40	89	11	59	56	7
MAL	10	28	49	0	26	13
NEB	103	0	15	46	46	0
NWR	41	0	10	76	4	4
OBB	14	0	0	34	39	17
SEB	14	0	11	0	2	52
SMB	62	20	49	76	5	6
TBB	111	0	0	1	20	1
TEB	71	3	0	25	24	1
TRB	37	2	33	35	25	13
Total	1,479	350	485	772	653	223

DNA was extracted using QIAamp 96 DNA QIAcube HT Kit (Qiagen, Toronto, Ontario, Canada) on a QIACube HT (Qiagen) following the manufacturer's protocol. Tissue samples were disrupted using a Tissue‐Lyser II (Qiagen) mixing 2 × 10 s at 20 s^−1^. DNA was eluted twice in 100 μl Buffer AE (Qiagen) preheated to 70°C. DNA extracts were quantified using QuantiT PicoGreen dsDNA Assay Kit (Thermo Fisher Scientific, Waltham, MA USA) and read on a FLUOStar OPTIMA fluorescence plate reader (BMG Labtech, Ortenberg, Germany). Individuals were genotyped using SNP Type assays (Fluidigm) following the manufacturer's protocols, targeting 95 SNPs previously established for the classification of farmed and wild salmon in Newfoundland (Wringe, Stanley, et al., 2018). At the applied posterior probability threshold (see below), this panel has been shown to assign individuals to a genetic class with over 90% accuracy, based on simulations in “hybriddetective” (Wringe, Stanley, Jeffery, Anderson, & Bradbury, 2017a; Wringe, Stanley, et al., 2018), with high congruency to genetic class assignment conducted using STRUCTURE (Pritchard, Stephens, & Donnelly, 2000; Sylvester et al., 2018). Each plate extraction included 10 redundant samples to detect processing errors. A total of 220, 190 and 214 samples from 2014, 2015 and 2016, respectively, were genotyped a second time to estimate the genotyping discordance rate which was used as a proxy for the genotyping error rate for each year (Pompanon, Bonin, Bellemain, & Taberlet, 2005).

### 2.2 Statistical analyses

All analyses were run and figures created using R v. 3.4.1, and data manipulation and conversion conducted using “genepopedit” (Stanley, Jeffery, Wringe, DiBacco, & Bradbury, 2017). Wild and farmed baseline individuals were simulated and centred (see Karlsson, Diserud, Moen, and Hindar (2014)) from the actual baseline samples using the R package “hybriddetective” (Wringe et al., 2017a) to reduce the erroneous interpretation of naturally occurring inter‐river genetic variation as evidence of introgression. Samples were classified into one of six genetic classes: pure wild, feral, first‐generation hybrids (F1), second‐generation hybrids (F2), backcross wild (BCW) or backcross feral/farmed (BCF) using NewHybrids (Anderson & Thompson, 2002). This approach implements a Bayesian Markov chain Monte Carlo approach for assignment, producing a posterior probability per class for each individual based on the provided baselines. NewHybrids was run using the R package “parallelnewhybrid” (Wringe, Stanley, Jeffery, Anderson, & Bradbury, 2017b) with a burn‐in of 50,000 and 100,000 sweeps. All samples were pooled together by year, with samples from each river run independently to reduce bias, such that naturally occurring genetic differentiation between rivers was not misinterpreted as signals of introgression. We then filtered individuals at a minimum posterior probability of assignment to a single class of 0.8 (Wringe, Jeffery, et al., 2018), resulting in 3,962 assigned individuals (see Table 2 for a breakdown of final sample size by age class). Per‐river class proportions were calculated for all parr, young‐of‐year (YoY) parr only and parr within a single cohort. Overall proportions were estimated after weighing by the axial length of each river (the distance along a straight line along the longest axis of the river; Porter, Riche, & Traverse, 1974) to reduce bias in sampled population size (Wringe, Jeffery, et al., 2018).

**Table 2:** Estimated relative fitness (and standard error of estimates across rivers) for the first two years of development (young‐of‐year (YoY) to 1+, 1+ to 2+) based on changes in population composition of genetic class

	Wild	F1	Feral
YoY to 1+	1 (0.09)	0.81 (0.26)	0.15 (0.11)
1 + to 2+	1 (0.10)	0.83 (0.42)	0.26 (0.24)

The relative fitness of wild, feral and first‐generation (F1) hybrids was estimated using a single cohort of individuals (2014 YoY, 2015 1+, 2016 2+) for each age/time step (YoY to 1+, 1+ to 2+). Relative fitness estimates of second‐generation hybrids (F2 and backcross) were not calculated due to restrictions in sample sizes. Traditional methods of estimating the relative fitness (or relative survival as a proxy of relative fitness; Hendry, 2017; Rice, 2004) of individuals of a known genotype (i.e., AA, Aa and aa) were applied to individuals of known genetic class (i.e., pure wild, F1 and pure feral; Thurman & Barrett, 2016). We calculated the proportional change in the population composition of a genetic class (*P_t+_*_1_/*P_t_*, where *P* is the class proportion at year *t*) within each river, then averaged across rivers and divided by the maximum proportional change (i.e., the average proportional change of the wild class) to obtain the relative, overall survivorship of each class across the region. Sites with fewer than 10 individuals per age class were removed from the calculation. Additionally, if the formula for the proportional change of a given genetic class at time *t* resulted in a denominator of 0, these rivers were removed for that time point calculation for that genetic class. This estimate of relative survivorship was interpreted as the relative fitness (w) of each genetic class.

### 2.3 Individual‐based modelling approach

We used an individual‐based salmon eco‐genetic model (IBSEM) developed by Castellani et al. (2015) to explore the possible long‐term effects of various invasion scenarios and relative survival associated with the farmed genotype in southern Newfoundland. IBSEM models the outcome of Atlantic salmon populations in response to invasion of domesticated individuals. Duration of invasion and recovery, wild population size and number of invaders, environmental conditions, individual size and genotypic and phenotypic differences between individuals of farm and wild origin are considered to model population changes in abundance, genotype and individual size. Growth and survival are simulated by stochastic procedures that are influenced by genotype, fish size and age, temperature and population density at three life stages: embryo, juvenile and adult. The effects of the genetic make‐up in the life history of the individuals are modelled through three independent sets of loci, one set for each life stage. The distribution of genetic effects across the 21 loci is modelled via an exponentially declining function, where the last locus has no effect and is used as a neutral marker. Through the influence of genotype, the differential between growth and survival of wild and feral individuals can be set and the consequences observed over time. Simulated loci are unlinked with possible gamete recombination and random inheritance (and are therefore influenced by drift), and a range of influences on phenotype and therefore suitability to the environment. The sum of the genetic effects is linearly related to phenotype, such that genotypic values approaching 1 are associated with growth and survival rates typical of wild salmon, and values approaching zero are associated with rates observed in farm escapees. Reproductive success of both wild and domestic individuals is sex‐specific, with female fertility dependent upon weight, and male reproductive success dependent upon length, with the possibility of precocial sexual maturation. Farm escapees are given a reduced spawning success than fish of any genetic make‐up that are born in the wild. We tested three temporal scenarios of invasion to investigate the impacts of consistent, annual invasion as a (a) short‐term, large escape event over 10 years of invasion relative to an (b) intermediate invasion rate (over 50 years) and (c) long‐term, trickle escapes (over 100 years; Table 3). For each temporal scenario, three levels of the magnitude of invasion were tested (no invasion, intermediate invasion and high invasion). Invasion levels were set such that the total number of invaders was equal across scenarios (i.e., 0, 500 and 1,000 invaders annually for 10 years; 0, 100 and 200 invaders annually for 50 years; and 0, 50 and 100 invaders annually for 100 years). Each temporal scenario and magnitude of invasion was tested at three levels of relative feral parr survival: our estimated value, low survival (half our estimate) or high survival (double our estimate). Our estimates of hybrid relative survival were not incorporated as IBSEM infers this based on additive genetic inheritance. It should be noted that the high survival scenario, while high relative to that estimated for southern Newfoundland populations, is still lower than most previous estimates of relative survival of feral parr (McGinnity et al., 2003, 1997). We compared the change in adult population abundance (both wild and escaped farmed fish) and sum of the genetic effects across the adult set of genes included in the simulation to observe changes in the genetic fitness of the population. All models were run for 100 years prior to invasion to ensure model stability and for 100 years after the invasion period ceased to assess time to recovery. All other parameters remained consistent across models. A full list of parameters, set to be representative of Newfoundland salmon and environmental conditions in the region (Veinott et al., 2018; correspondence with Dr. Brian Dempson) or set as default, can be found in the Supporting Information. Two parameters reflective of overall wild survival were selected by trial‐and‐error to achieve a consistent (stable) population size under a zero invasion scenario with all other parameters set as described in Supporting Information Table S1.

**Table 3:** Scenarios tested in an individual‐based salmon eco‐genetic modelling (IBSEM) approach (Castellani et al., 2015). All other parameters were consistent across scenarios and can be found in the Supporting Information. Each of the three values for number of invaders was modelled using each pair of relative survival parameters (low, calculated and high), resulting in nine models for each temporal scenario (see Figures 3 and 4)

Temporal scenario	Invasion time (years)	Number of invaders annually	Relative survival (parr0/parr1)
Scenario 1: Short‐term	10	0, 500, 1,000	0.075/0.13, 0.15/0.26, 0.3/0.52
Scenario 2: Intermediate	50	0, 100, 200	0.075/0.13, 0.15/0.26, 0.3/0.52
Scenario 3: Long‐term	100	0, 50, 100	0.075/0.13, 0.15/0.26, 0.3/0.52

## 3 RESULTS

**Figure 2:**
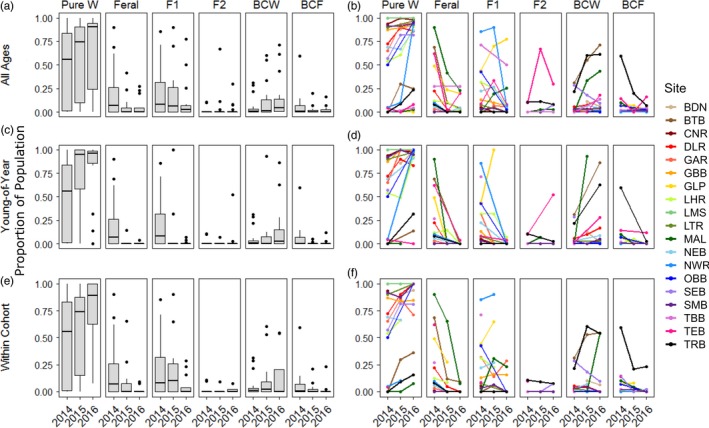
Per cent population composition by genetic class (pure feral, pure wild, F1, F2, backcross wild (BCW) and backcross feral (BCF)) across three sampled years including (row 1) individuals of all ages, (row 2) young‐of‐year (YoY) only and (row 3) within cohort (2014 YoY, 2015 1+, 2016 2+). Each panel comprises a single genetic class, including (a, c, e) boxplots of overall trends and (b, d, f) individual river proportion indicated by colour. Sites with fewer than 5 assigned samples were removed to avoid bias in river composition. Temporal fluctuations in within‐cohort population composition were used to estimate relative survival as a proxy of relative fitness for pure wild, feral and F1 genetic classes (see Methods)

**Figure 3:**
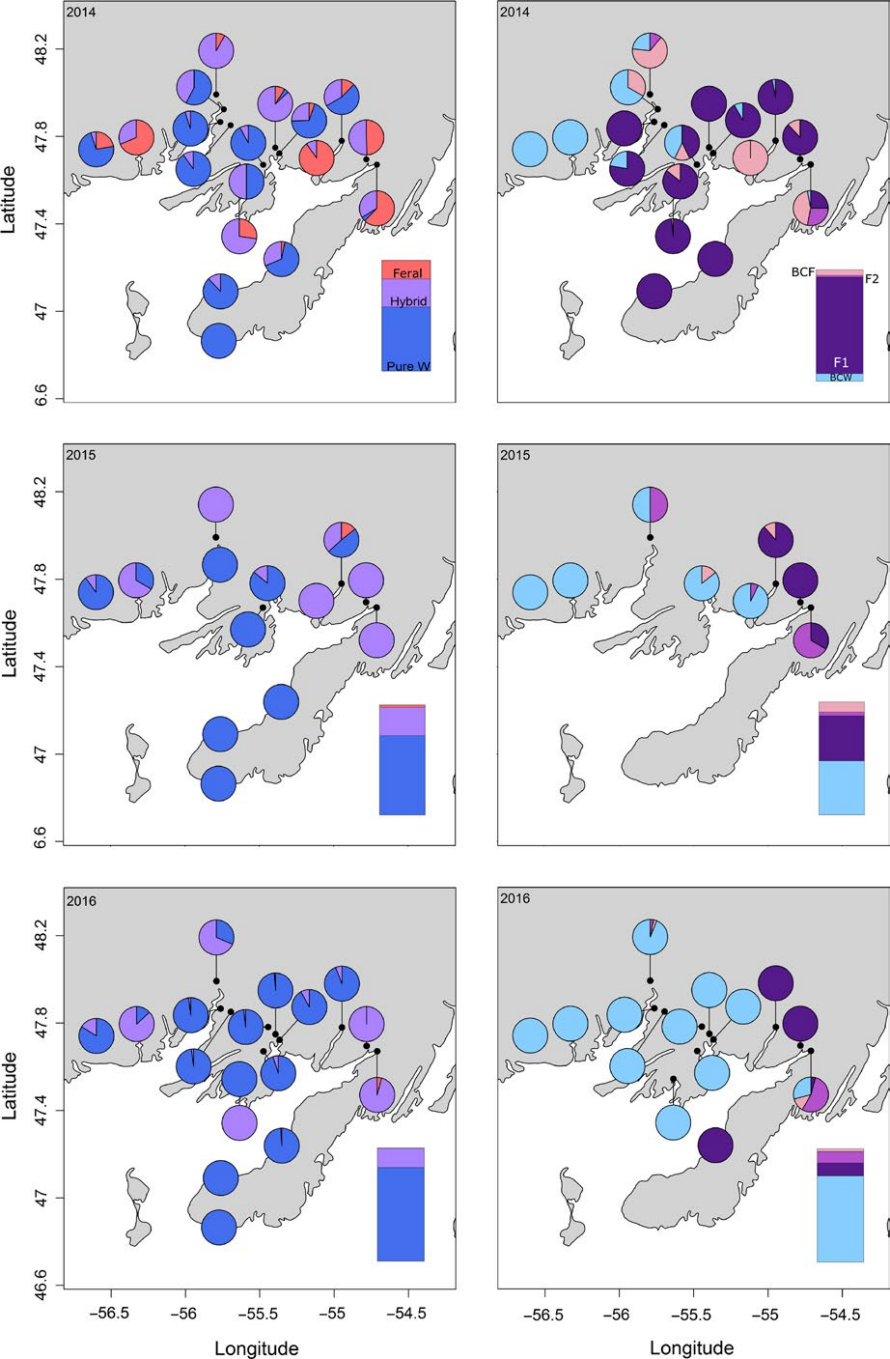
Genetic class (pure wild, pure feral, F1, F2, backcross wild (BCW) and backcross feral (BCF)) proportion for each sampled river as determined using NewHybrids for young‐of‐year (YoY) samples across all sampled years. Panels in column one convey proportions of wild, feral and hybrid parr (all hybrid classes combined) while panels in column two convey proportions of hybrid classes (F1, F2, backcross wild (BCW), backcross feral (BCF)) for each river with hybrid individuals detected in that year (row), as indicated in purple in column one. Bars in each panel represent overall proportions after standardizing by river size (axial length). Corresponding figures for other age classes can be found in Supporting Information Figure S1

A total of 4,619 parr were genotyped using the SNP panel. The genotype error rate was estimated to be 0.17%, 0.01% and 0.13% for 2014, 2015 and 2016, respectively. Of all samples, 86% of individuals were classified by NewHybrids above the posterior probability threshold of 0.8. Across age classes, pure wild parr were the most prevalent class, followed by hybrids and feral parr (Figure 2a,b), with few exceptions in particular rivers. After scaling by river size (axial length), wild population proportion increased overall (increasing by a factor of 1.05 and 1.16 in the first and second year, respectively), with a corresponding decline in feral (by a factor of 0.62, 0.33) and hybrid parr (by a factor of 0.93, 0.57; Figure 2). First‐generation hybrids (F1) were the most common hybrid class in 2014, with a steady decline in most rivers (Figures 2 and 3) in subsequent years (by a factor of 0.68 and 0.25 in the first and second year, respectively). Population proportion of backcross wild (BCW) parr increased during the first year (by a factor of 4.8), driven mostly by dramatic increases in BCW proportion in three rivers, MAL, BTB and TRB. BCW proportion remained generally constant (population proportion decreased by a factor of 0.91) in the second year of life. These trends were consistent within young‐of‐year (YoY) parr (Figures 2c,d and 3) and within a single cohort (Figure 2e,f). Increasing class proportions within a cohort suggest a higher relative fitness compared with those classes that are observed to decrease with time. We applied this reasoning to estimate relative survival as a proxy of fitness and strength of selection against classes that are seen to decrease over time, relative to wild types within a single cohort. As such, the relative fitness of the wild class was 1 for all estimates. Relative fitness was higher for F1 than for feral salmon and was slightly lower for both classes in the first year of development than the second year (Table 2). Variance (reported as standard error) in the relative survival of F1 parr was considerably higher than that of feral or wild individuals (Table 2). Although low within‐river sample sizes at single time points limited our ability to estimate river‐specific relative survival of genetic classes, we report these estimates in Supporting Information Table S2 to demonstrate variance in estimated relative survival across rivers.

**Figure 4:**
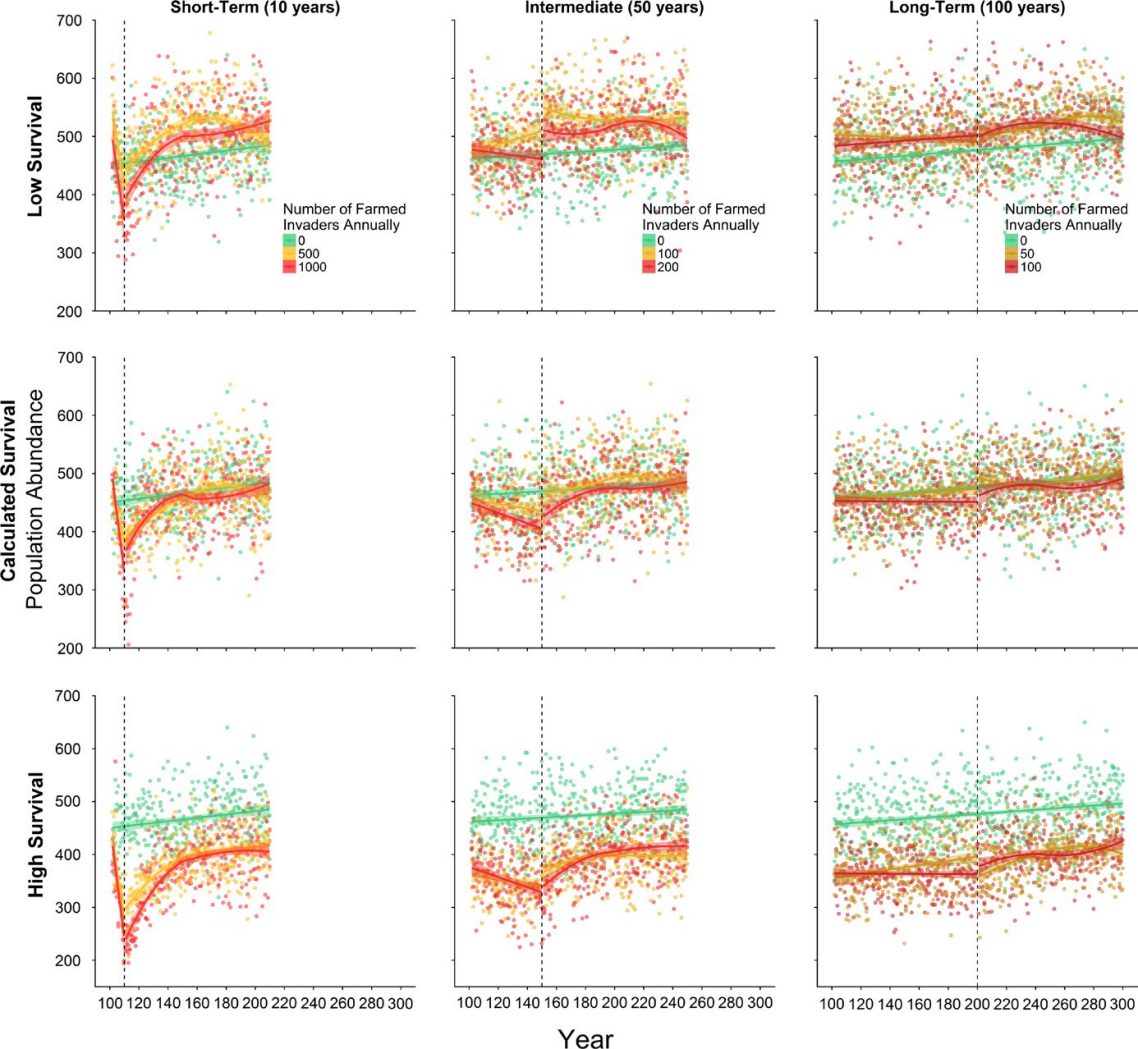
Adult population abundance as estimated using IBSEM (Castellani et al., 2015) for all tested scenarios (see Table 3). Three invasion scenarios (columns: short‐term, intermediate and long‐term) were each modelled at three levels of relative survival for feral parr (rows: half calculated relative survival, calculated relative survival for the study region (as shown in Table 2) and double calculated relative survival). Each of these nine scenarios was tested with three levels of invasion (number of farmed invaders) as indicated by colour. Invasion started after 100 years of settling; the time at which invasion ceased (duration of invasion) is indicated by a vertical dashed line. Loess curves are used for visualization of trends in the data

**Figure 5:**
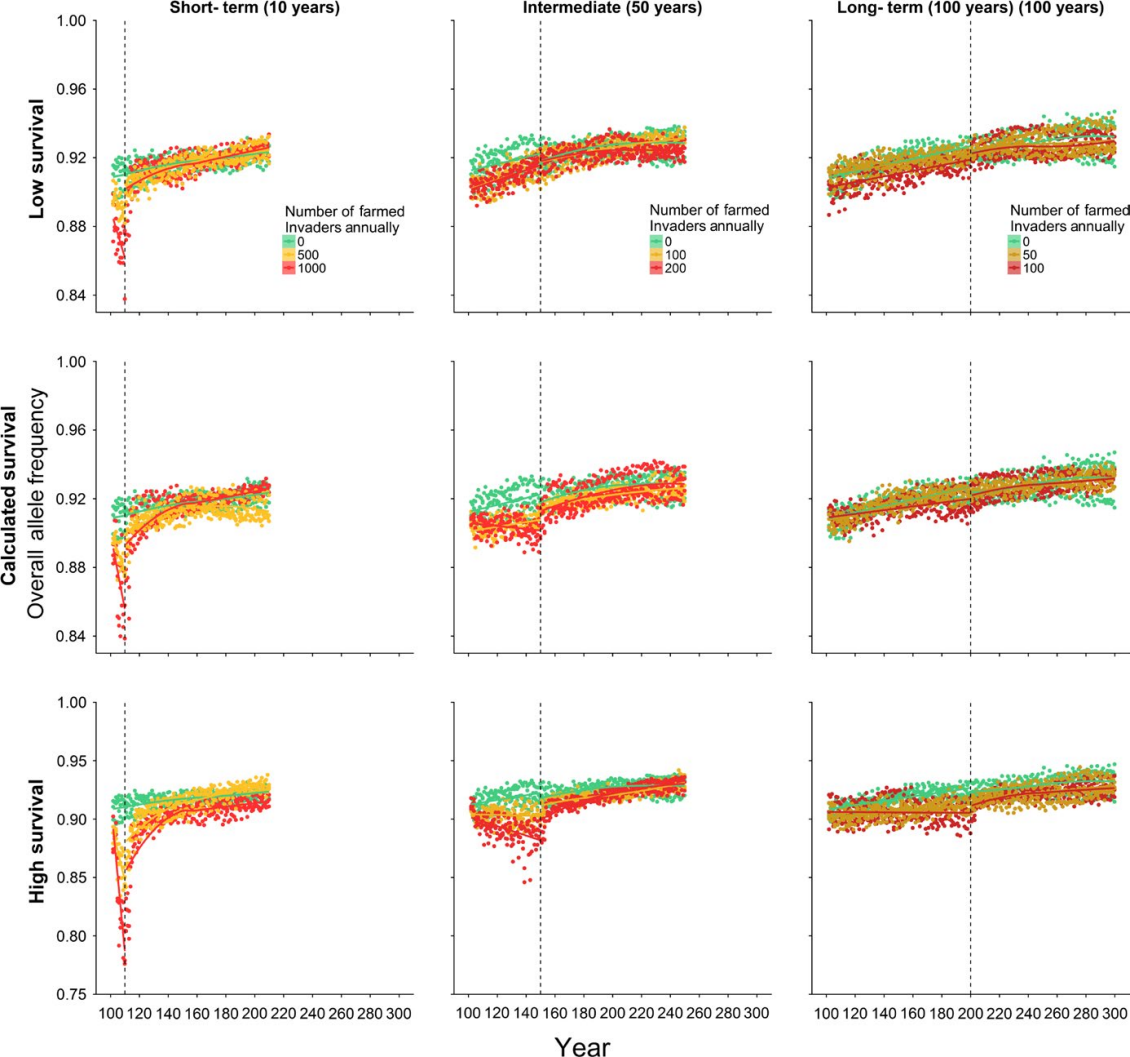
Average allele frequency across 21 simulated genotypes as estimated using IBSEM (Castellani et al., 2015) for all tested scenarios (see Table 3). Three invasion scenarios (columns: short‐term, intermediate and long‐term) modelled at three levels of relative survival for feral parr (rows: half calculated relative survival, calculated relative survival for the study region (as shown in Table 2) and double calculated relative survival). Each of these nine scenarios was tested with three levels of invasion (number of farmed invaders) as indicated by colour. Invasion started after 100 years of settling; the time at which invasion ceased (duration of invasion) is indicated by a vertical dashed line. Loess curves are used for visualization of trends in the data

Our estimates of average relative survival of individuals with feral genotypes (see Table 2) were incorporated into the individual‐based modelling approach (IBSEM). We examined three temporal scenarios (Table 3), and three relative survival scenarios: our calculated relative survival, half and twice that value. These scenarios revealed differences in population response and recovery, affirming the importance of estimating relative survival in predicting population response to invasion. Severity of the population crash and time to recovery increased with increasing relative survival of feral parr and decreasing duration or increasing intensity of invasion (Figure 4). In calculated relative survival models, full recovery was observed after 30–40 years post‐invasion in the short‐term invasion scenario, less than 20 years in the intermediate scenario and immediately after invasion ceased in the low invasion scenario. High relative survival of farmed invaders and the short‐term temporal scenario resulted in the greatest decrease in overall population abundance, to as few as 200 individuals after 10 years of invasion, from a stable population of approximately 475 under a zero invasion scenario (Figure 4). Additionally, high relative survival tests did not fully recover after 100 years in any of the invasion scenarios. Overall, modelled allele frequencies shifted towards the farmed genotype in all temporal and survivorship scenarios following similar patterns as population abundance. That is, the severity of the change in allele frequency increased with increasing relative survival of feral parr and increasing intensity (decreasing duration) of invasion (Figure 5). Time to recover to allele frequencies comparable with the zero invasion models were similar and rapid across all scenarios, with the longest recovery time observed at approximately 50 years after ceasing invasion in short‐term invasion models at high relative survival.

## 4 DISCUSSION

With the continued growth of Atlantic salmon aquaculture, understanding and predicting the impacts of escape events of farmed Atlantic salmon are central to the persistence of wild populations across the species range (Forseth et al., 2017; Glover et al., 2017). The survival and fitness of farmed escapee Atlantic salmon relative to the wild populations they invade has the potential to ultimately determine the genetic impacts of invasion on wild populations, yet population‐ or region‐specific relative survival of individuals of aquaculture ancestry in the wild is rarely estimated. Here, we build on previous work investigating the extent of hybridization following a large escape event in southern Newfoundland (Wringe, Jeffery, et al., 2018) and calculate relative survival and associated strength of selection against feral and hybrid parr using temporal changes in population composition. We demonstrate decreased survival of offspring of aquaculture escapees relative to pure wild individuals and explore how relative survival and rates of invasion may impact wild populations using an individual‐based modelling approach (Castellani et al., 2015). This method of estimating survival and consequential long‐term genetic impacts of farmed invasion provides a novel field‐based approach that has previously generally been limited to controlled experiments that do not account for population or regional variation. By modelling various invasion and survival scenarios, we highlight the importance of considering region‐specific relative survival when predicting population trajectories under various rates of invasion with the potential to inform approaches to population conservation and fisheries management.

We examined temporal changes in the proportion of hybrids within young‐of‐year (YoY) samples and within a single cohort to illuminate the factors influencing the presence and survival of farmed escaped offspring in the wild. Temporal changes in hybrid proportions within a cohort reflected continued declines in hybrids and feral parr with an increase in wild‐type individuals over time as noted elsewhere (DFO, 2018; McGinnity et al., 2003, 1997; Skaala et al., 2012). Changes in the relative proportions of hybrid types also followed a similar pattern with a relative decrease in F1s and an increase in backcross wild individuals. This change in population composition over time suggests reduced relative fitness of feral and hybrid offspring compared to pure wild. Interestingly, our estimates of relative survival are considerably lower than some previous estimates for feral parr (ranging from 0.61 to 1.53, relative to pure wild survival of 1; Hindar, Fleming, McGinnity, & Diserud, 2006) but comparable to that of previous studies for F1 parr (Fleming et al., 2000; McGinnity et al., 2003, 1997; Skaala et al., 2012). Relative survival of feral individuals in the wild is likely related to the degree of genetic differentiation between wild and farmed strains. Our low estimates of feral survival may thus reflect high domestication selection or drift in domestic salmon (Glover et al., 2017; Gross, 1998) or pre‐existing genetic differences between wild Newfoundland populations and the Saint John River lineage currently stocked in the region (Bradbury et al., 2015; Moore et al., 2014). Consequential genetic divergence may result in reduced suitability to conditions in southern Newfoundland (Vandersteen, Biro, Harris, & Devlin, 2011). Our observations of an increase in the proportion of wild backcrossed individuals (BCW) in some populations suggest comparable survival to pure wild individuals, consistent with previous findings of higher performance in backcross classes (Fraser, Cook, Eddington, Bentzen, & Hutchings, 2008; McGinnity et al., 2003, 1997).

In 2014, the year immediately following a large escape event, levels of hybridization were consistent with reported impacts elsewhere (Glover et al., 2017; Karlsson et al., 2016), with all but one of 18 rivers showing evidence of hybrid or feral parr presence. In subsequent years, the overall proportion of hybrids and of F1 hybrids decreased, suggesting that mating between farmed escapees and wild salmon was highest immediately following the escape, likely due to the large influx of farmed individuals. This decrease is consistent with reduced contributions from this escape event over time. The presence of second‐generation hybrids throughout our sampling years indicates that hybrid individuals and therefore farmed invaders unrelated to the escape event are present in these rivers, indicative of continued low‐level trickle invasion (Wringe, Stanley, et al., 2018). While general temporal trends are consistent across rivers, there is a large degree of spatial variation in genetic class proportion at a given time point, as previously reported by Sylvester et al. (2018), with consequential variation in relative survival estimates. Due to low within‐river sample size at individual time points, we focus on average relative survival of parr in the sampled region. However, the approach for estimating relative survival applied here can be easily applied for river‐scale estimates of relative survival when sample sizes are adequate.

The precocious maturation of hybrid or feral male parr may influence levels of hybridization and introgression and thus alter allele frequency (Gjerde, Simianer, & Refstie, 1994) and lower productivity. This phenomenon may increase the relative fitness of feral parr as evidence suggests that reproductive success of farmed precocious males may be higher than that of wild individuals (Garant, Fleming Ian, Einum, & Bernatchez, 2003). Genetic introgression may be exacerbated by feral precocial males contributing to the population as allele frequency shifts towards the farmed genotype to a greater extent in early life stages (Castellani et al., 2015). Although aquaculture breeding practices often select against early maturation, early maturation is also largely environmentally determined (Good & Davidson, 2016; Jonsson, Jonsson, & Finstad Anders, 2012), and high rates of sexual precocity have been reported in wild southern Newfoundland populations (Dalley, Andrews, & Green, 1983; Myers, 1984). However, common garden experiments have revealed male parr maturation to be lower in farmed progeny than in wild parr (McGinnity et al., 2007), with F1 hybrids demonstrating an intermediate likelihood of precocial maturation. Estimates of rates of hybrid precocial maturation and fitness in the region would enhance the ability to predict rates of introgression between and wild and farmed salmon.

Our modelling results suggest that consequences of invasion of farmed salmon could vary dramatically with the magnitude and temporal scope of escape events. Repeated large pulses of invasion were more detrimental to wild population productivity than continued low‐level escape events. Interestingly, previous modelling efforts have disagreed on the relative impact of low‐level chronic or large pulse escape events. Hindar et al. (2006) and Hindar and Diserud (2007) suggest greater impacts following large pulses of escapees contrasting the results of Baskett et al. (2013) who suggest that low‐level leakage may be more detrimental to wild populations due to a gradual shift towards the farmed genotype. This variation in results has been suggested to be due to the time period considered and equilibrium status of model simulations (Baskett et al., 2013). However, the temporal scenarios modelled here made very little difference to long‐term population trajectories compared to the impact of the parr survivorship parameters. Under the most extreme scenarios (i.e., high relative survival), wild population abundance did not fully recover regardless of the temporal scenario even after 100 years of recovery, although overall genetic effects were not substantially different after 100 years of recovery, suggesting that recovery in population abundance is limited despite shifts in allele frequency towards the wild type after invasion has ceased. Decreasing population abundance with increasing relative survival of feral parr is likely due to an overall reduction in population productivity as a consequence of higher feral and hybrid presence and thus contribution to the gene pool, compared to models with lower relative survival of feral parr. In the models with our calculated relative survival rates for southern Newfoundland, population abundance and allele frequency recovered shortly after invasion ceased. In reality, however, southern Newfoundland wild populations continue to decline (COSEWIC, 2011; DFO, 2013). This suggests that farmed invasion may be ongoing or that other factors such as at‐sea survival, habitat degradation or fishing pressures may be at play (Bourret et al., 2011; Vähä, Erkinaro, Niemelä, & Primmer, 2007), exacerbating large‐scale population declines.

Although IBSEM is a comprehensive Atlantic salmon individual‐based model, there are additional factors such as the introduction of disease, fishing pressure and river flow that may influence population response to invasion (Castellani et al., 2015). IBSEM implements a constant or random number or proportion of farmed invaders, and constant relative survival and reproduction. Consequently, more realistic escape scenarios such as constant low‐level invasion combined with a large, single‐year event or multiple invasions at infrequent intervals are not currently considered, possibly limiting our understanding of population response to domestic invasion. Relative survival in IBSEM is generally reflective of marine survival, as this is known to strongly influence overall survival rates (Jonsson, Jonsson, & Hansen, 2003; McGinnity et al., 2003). However, although we have attempted to parameterize to reflect conditions in southern Newfoundland, a paucity of available region‐specific data (such as relative marine survival) may reduce the accuracy of these models. With ongoing investigations within the region, estimates of marine return may be informed by subsequent sampling, allowing for future modifications and improvements to these simulations. Despite the current limitations, simulations such as those conducted here allow an unprecedented opportunity to explore long‐term population responses to invasion of farmed escaped salmon and can directly inform decisions regarding management practices and the conservation of wild populations.

Extending our survival estimates with the inclusion of numerous cohorts would provide additional support for our estimates; however, sample sizes of 2015 YoY individuals were insufficient to include this cohort in our analysis. Also, limiting the analysis to only the highly supported hybrid assignments by filtering individuals by posterior probability in NewHybrids may bias our results for some hybrid classes as individuals that do not reach this threshold are more likely to be second‐generation hybrids, backcrosses or further introgressed individuals (Sylvester et al., 2018). However, as this bias is consistent across years, we expect temporal fluctuations in hybrid classes to be robust and with little to no effect on our parameter estimates as we did not estimate relative survival of second‐generation hybrid classes.

Existing efforts to estimate relative fitness and, accordingly, strength of selection against feral or hybrid parr in wild Atlantic salmon populations invaded by farmed escapees are often labour‐intensive, requiring experimental manipulation in the laboratory or in rivers, and do not consider how variation in landscape and susceptibility of a wild population to introgression may differentially impact survival of individuals of aquaculture ancestry. We present a novel approach utilizing genetic data following a large escape event to classify individuals to a genetic class (pure wild, pure feral, F1, F2, BCW, BCF) and infer relative fitness based on within‐cohort changes to class composition, applied to a region of southern Newfoundland. These approaches may be easily applied at any scale with sufficient sampling. We further apply our estimates to demonstrate that survival of feral parr, relative to their wild counterparts, affects long‐term levels of introgression, particularly under stochastic invasion conditions. Wild population abundance was greatly affected by the relative survival of feral parr without full recovery in all invasion scenarios (short‐term, intermediate and long‐term) at high relative survival of feral parr. These results indicate the importance of obtaining accurate estimates of region‐ or population‐specific relative fitness to predict population response to farmed invasion. Incorporating this knowledge may allow a deeper understanding of possible impacts on wild populations and may inform management and conservation decisions accordingly.

## CONFLICT OF INTEREST

None declared.

## DATA AVAILABILITY

Data for this study are available at the Dryad Digital Repository: Genotype data of 2014 and 2015 samples can be found at https://doi.org/10.5061/dryad.3k888n7. Genotype data of 2016 samples and field ages assigned to all individuals can be found at https://doi.org/10.5061/dryad.2kc5rh0.
