# Supplementary material for: Estimating the relative fitness of escaped farmed salmon offspring in the wild and modelling the consequences of invasion for wild populations
Source: Evol Appl. 2019 Jan 28;12(4):705–17. doi: 10.1111/eva.12746 (PMC6439497; doi:10.1111/eva.12746)
Supplement: Supplementary file 1 [file EVA-12-705-s001.docx]

**Supplemental Information for:**

**Estimating the relative fitness of escaped farmed salmon offspring in the wild and modeling the consequences of invasion for wild populations**

| Table S1: Parameter inputs used for running IBSEM. Parameters were partially informed by previous estimates of conditions in southern Newfoundland (i.e. temperature and size estimates) (Veinott et al., 2018; correspondence with Dr. Brian Dempson), or set to default. Parameters in bold are those tested at multiple levels in the present study (see Table 3). Survival parameters (indicated by *) were determined by trial-and-error to achieve a consistent population size under a zero-invasion scenario with all other parameters set. | | |
| --- | --- | --- |
| File | Value | Description |
| Adult | 4.6 | State of "activity" (Piou and Prevost, 2012) for first year (adult1SW) here used to model intra population (in wild and farm) and inter-population (between wild and farm) differences. It depends on genes. |
|  | 2.2 | State of "activity" (Piou and Prevost, 2012) for second and further years (adult>1SW), here used to model intra-population (in wild and farm) and inter-population (between wild and farm) differences. It depends on genes. |
|  | 1.2 | Differential in activity between farm and wild fish farm=survivalDelta*wild. It depends on genes. |
|  | 0.31 | Allometric relationship between growth rate and fish mass (Forseth et al. 2001) |
|  | 0.33 | Growth speed according to temperature (Forseth et al. 2001) |
|  | 0.2 | Parameter regulating optimal temperature for growth (Forseth et al. 2001) |
|  | 2 | Lower critical water temperature for growth (Forseth et al. 2001) |
|  | 20 | Higher critical water temperature for growth (Forseth et al. 2001) |
|  | 750* | Survival parameter taken from (Piou and Prevost, 2012). It is a multiplicative term that links mortality to length. |
|  | -2.5* | Survival parameter taken from (Piou and Prevost, 2012). It sets the exponential dependence of mortality from length. |
|  | 0.6 | Differential on survival chances between farm and wild fish farm=survivalDelta*wild. It depends on genes. |
|  | 0.1 | Standard deviation from mean of adult weight (fraction of mean) |
|  | 0.99/1/1 | Maturation probabilities for 1SW, 2SW, and 3SW wild adults |
|  | 0.25/0.47/1 | Differential in maturation probabilities (1SW, 2SW, and 3SW) between farm and wild fish farm=probabilityDelta*wild |
|  | 0.03/0.4 | Reproductive success of farm salmon compared to wild (males, females) |
| Embryo | 1.6345 | Parameter defining alevin size |
|  | 12.991 | Parameter defining alevin size |
|  | 0.035 | Standard deviation from mean of alevin length (fraction of mean) |
|  | -3.16 | Parameter that affects slope in weight-dependent mortality rate calculation. |
|  | 1.12 | Parameter that affects offset in weight-dependent mortality rate calculation. |
|  | 0.7 | Differential in survival rates between farm and wild fish farm=survivalDelta*wild. It depends on genes. |
| Parr0 | 0.75 | State of "activity" (Piou and Prevost, 2012), here used to model intra-population (in wild and farm) and inter-population (between wild and farm) differences. It depends on genes. |
|  | 1.3 | Differential in activity between farm and wild fish farm=activityDelta*wild. It depends on genes. |
|  | 0.31 | Allometric relationship between growth rate and fish mass (Forseth et al. 2001) |
|  | 0.374 | Growth speed according to temperature (Forseth et al. 2001) |
|  | 0.201 | Parameter regulating optimal temperature for growth (Forseth et al. 2001) |
|  | 7 | Lower critical water temperature for growth (Forseth et al. 2001) |
|  | 24 | Higher critical water temperature for growth (Forseth et al. 2001) |
|  | 0.983 | Daily survival rate at infinitely high fry density during the growth (warm) season (May-Nov). The precise value for each individual depends on genes. |
|  | 0.9988 | Daily survival rate at infinitely high fry density outside the growth (cold) season (Dec-Feb). The precise value for each individual depends on genes. |
|  | **0.15** | **Differential in survival rates between farm and wild fish farm=survivalDelta*wild. It depends on genes.** |
|  | 0.25 | Standard deviation from mean of fry weight (fraction of mean) |
|  | 0.13 | Parameter determining the steepness of the fry maturation probability curve. |
|  | 107 | Parameter determining the horizontal shift of the fry maturation probability curve. |
|  | 100 | Parameter determining the maximum maturation probability for the fry maturation probability curve. |
|  | 0.2 | Parameter determining the steepness of the fry smolting probability curve. |
|  | 103 | Parameter determining the horizontal shift of the fry smolting probability curve. |
|  | 100 | Parameter determining the maximum maturation smolting for the fry smolting probability curve. |
| Parr1 | 0.75 | State of "activity" (Piou and Prevost, 2012) for second and further years (parr >1+), here used to model intra-population (in wild and farm) and inter-population (between wild and farm) differences. It depends on genes. |
|  | 0.75 | State of "activity" (Piou and Prevost, 2012), here used to model intra-population (in wild and farm) and inter-population (between wild and farm) differences. It depends on genes. |
|  | 1.3 | Differential in activity between farm and wild fish farm=activityDelta*wild. It depends on genes. |
|  | 0.31 | Allometric relationship between growth rate and fish mass (Forseth et al. 2001) |
|  | 0.374 | Growth speed according to temperature (Forseth et al. 2001) |
|  | 0.201 | Parameter regulating optimal temperature for growth (Forseth et al. 2001) |
|  | 7 | Lower critical water temperature for growth (Forseth et al. 2001) |
|  | 24 | Higher critical water temperature for growth (Forseth et al. 2001) |
|  | 0.983 | Daily survival rate at infinitely high fry density during the growth (warm) season (May-Nov). The precise value for each individual depends on genes. |
|  | 0.9988 | Daily survival rate at infinitely high fry density outside the growth (cold) season (Dec-Feb). The precise value for each individual depends on genes. |
|  | **0.26** | **Differential in survival rates between farm and wild fish farm=survivalDelta*wild. It depends on genes.** |
|  | 0.25 | Standard deviation from mean of fry weight (fraction of mean) |
|  | 0.13 | Parameter determining the steepness of the fry maturation probability curve. |
|  | 107 | Parameter determining the horizontal shift of the fry maturation probability curve. |
|  | 100 | Parameter determining the maximum maturation probability for the fry maturation probability curve. |
|  | 0.2 | Parameter determining the steepness of the fry smolting probability curve. |
|  | 103 | Parameter determining the horizontal shift of the fry smolting probability curve. |
|  | 100 | Parameter determining the maximum maturation smolting for the fry smolting probability curve. |
| Smolt | 0.7 | State of "activity" (Piou and Prevost, 2012) for fish that smolted at the end of the first year of life (smolt 1+), here used to model intra-population (in wild and farm) and inter-population (between wild and farm) differences. It depends on genes. |
|  | 0.6 | State of "activity" (Piou and Prevost, 2012) for fish that smolted at the end of the second or more year of life (smolt >1+), here used to model intra-population (in wild and farm) and inter-population (between wild and farm) differences. It depends on genes. |
|  | 2 | Differential in activity between farm and wild fish farm=activityDelta*wild. It depends on genes. |
|  | 0.31 | Allometric relationship between growth rate and fish mass (Forseth et al. 2001) |
|  | 0.374 | Growth speed according to temperature (Forseth et al. 2001) |
|  | 0.201 | Parameter regulating optimal temperature for growth (Forseth et al. 2001) |
|  | 0 | Lower critical water temperature for growth (Forseth et al. 2001) |
|  | 24 | Higher critical water temperature for growth (Forseth et al. 2001) |
|  | 0.999 | Daily survival rate at infinitely high smolt density. The precise value for each individual depends on genes. |
|  | 0.75 | Differential in survival rates between farm and wild fish farm=survivalDelta*wild. It depends on genes. |
|  | 0.09 | Standard deviation from mean of smolt weight (fraction of mean) |
| Temp  (River/Sea) | 1.4/0.66 | January |
|  | 1.51/-0.27 | February |
|  | 2.1/-0.23 | March |
|  | 4.67/0.84 | April |
|  | 11.73/3.04 | May |
|  | 16.25/6.68 | June |
|  | 20.79/11.05 | July |
|  | 20.54/13.63 | August |
|  | 16.57/11.9 | September |
|  | 10.35/8.26 | October |
|  | 6.05/4.79 | November |
|  | 2.55/2.42 | December |
| Sim | **10-100** | **Number of years** |
|  | 100 | Years the populations is let to settle before beginning monitoring |
|  | 100 | Years the population is let to recover after introgression |
|  | 1 | Variables may be sampled for monitoring every n years |
|  | 30397 | Size of initial embryo population |
|  | 10000 | Size of initial fry population |
|  | 2000 | Size of initial parr1 population |
|  | 1000 | Size of initial parr2 population |
|  | 200 | Size of initial smolt population |
|  | 500 | Size of initial adult population |
|  | 6.1 | Diameter (mm) of the initial population of eggs |
|  | 23 | Fork length (mm) of the initial population of fry |
|  | 65 | Fork length (mm) of the initial population of parr1 |
|  | 100 | Fork length (mm) of the initial population of parr2 |
|  | 149 | Fork length (mm) of the initial population of smolts |
|  | 526 | Fork length (mm) of the initial population of smolts |
|  | 21 | Number of loci per chromosome |
|  | 0.15 | Parameter defining the steepness of the exponential distribution of genetic effects |
|  | Wild | Kind of initial population |
|  | Fixed | Incoming strayers can be a fraction of the population of a fixed number |
|  | 0 | Fraction of farm fish added to the returning population |
|  | **0-1000** | **Number of farm fish added to the returning population (if fixed numbed is used)** |
|  | 0.9 | Frequency of ‘wild’ (1) genes in the genotype of an average wild salmon |
|  | 0.1 | Frequency of ‘wild’ (1) genes in the genotype of an average farm salmon |
|  | 0.8 | Frequency of ‘wild’ (1) genes in the genotype of an average strayer |
|  | True | Flag to allow sneaker parr |
|  | False | Flag indicating whether level introgression is randomised |
|  | 0 | If introgression is randomised, this is the max variation |

| Table S2: Standardized river-specific strength of selection or fitness estimates for genetic classes pure wild, F1 and feral, for all rivers with sufficient sample size (>5) at each age class (2014 YoY to 2015 1+ and 2015 1+ to 2016 2+). Blank cells (-) are indicative of no presence of that class at the earlier time point within a river. | | | | |
| --- | --- | --- | --- | --- |
| Age Class | River | Wild | F1 | Feral |
| YoY to 1+ | DLR | 1 | - | 0.18 |
|  | GAR | 1 | 0.33 | 0 |
|  | GBB | 0.77 | 1 | - |
|  | GLP | - | 1 | 0.38 |
|  | LHR | 1 | 0.58 | 0.56 |
|  | LMS | 1 | - | - |
|  | NEB | 0.81 | 1 | 0 |
|  | NWR | 1 | 0.51 | 0 |
|  | SEB | 1 | - | - |
|  | SMB | 0.50 | 1 | - |
| 1+ to 2+ | BDN | 1 | - | - |
|  | BTB | 1 | 0 | 0.625 |
|  | DLR | 1 | - | 0 |
|  | GAR | 0.41 | 1 | - |
|  | GBB | 1 | 0.95 | - |
|  | LMS | 1 | - | - |
|  | MAL | - | 1 | 0.14 |
|  | SEB | 1 | - | - |
|  | SMB | 1 | 0 | - |
|  | TRB | 1 | - | - |


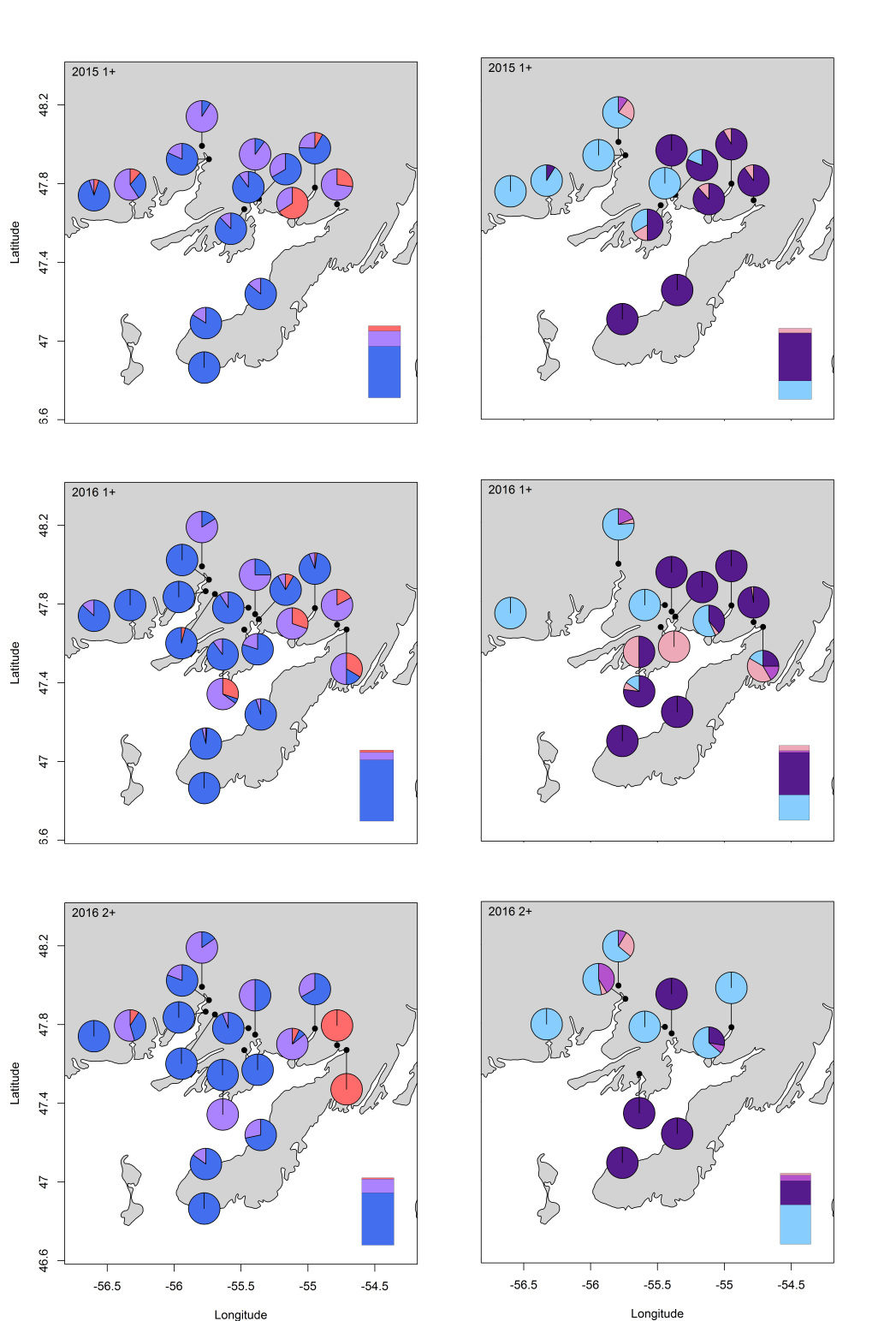


Figure S1: River specific proportions of genetic classes as determined using New Hybrids for 1+ and 2+ parr. Panels in column two convey proportions of hybrid classes (F1, F2, backcross wild (BCW), backcross feral (BCF)) for each river with hybrid individuals detected in that year (row), as indicated in purple in column one. Bars in each panel represent overall proportions after standardizing by river size (axial length), following colours indicated in Figure 3.
